# Supplementary material for: Identification of Genomic Regions Associated with High Grain Zn Content in Polished Rice Using Genotyping-by-Sequencing (GBS)
Source: Plants (Basel). 2022 Dec 28;12(1):144. doi: 10.3390/plants12010144 (PMC9824299; doi:10.3390/plants12010144)

Supplementary Figure S1: Frequency distribution of 92 RILs for FeBR- Iron content in Brown Rice (ppm); FePR- Iron content in Polished Rice (ppm); ZnBR- Zinc content in Brown Rice (ppm); ZnPR- Zinc content in Polished Rice (ppm) for all the four seasons

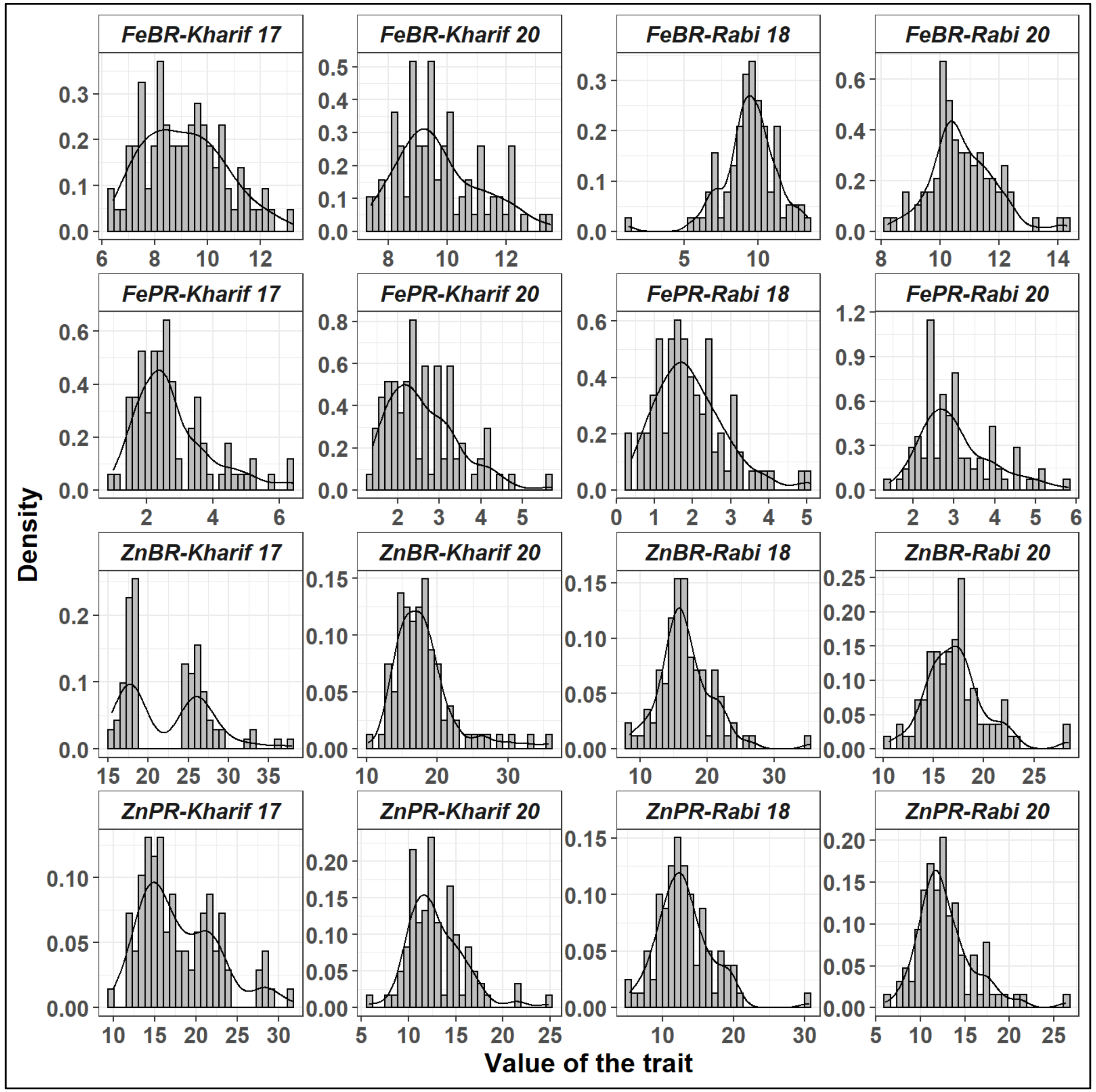

Supplementary Figure S2 : Frequency distribution of 92 RILs for DFF-Days to fifty percent flowering (days); NT-Number of Tillers per plant; PH- Plant Height (cm); PL- Panicle Length (cm); SPY- Single plant yield (gm) for all the four seasons

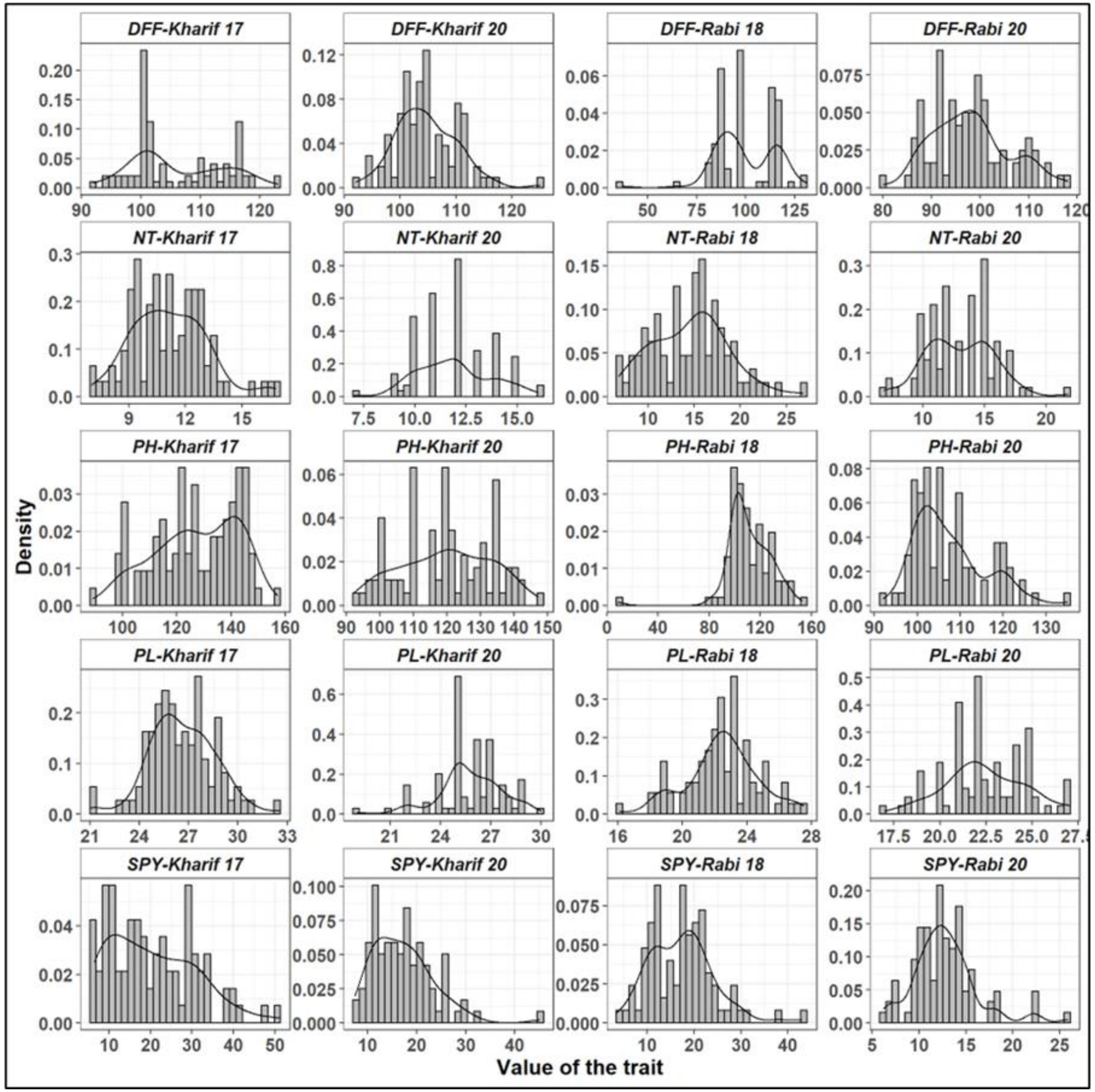

Supplementary Figure S3: Identified candidate genes in the QTL regions (qZnBR.2.1, qZnBR.9.2, qZnPR.2.1, qZnPR.6.2) by using WEGO analysis

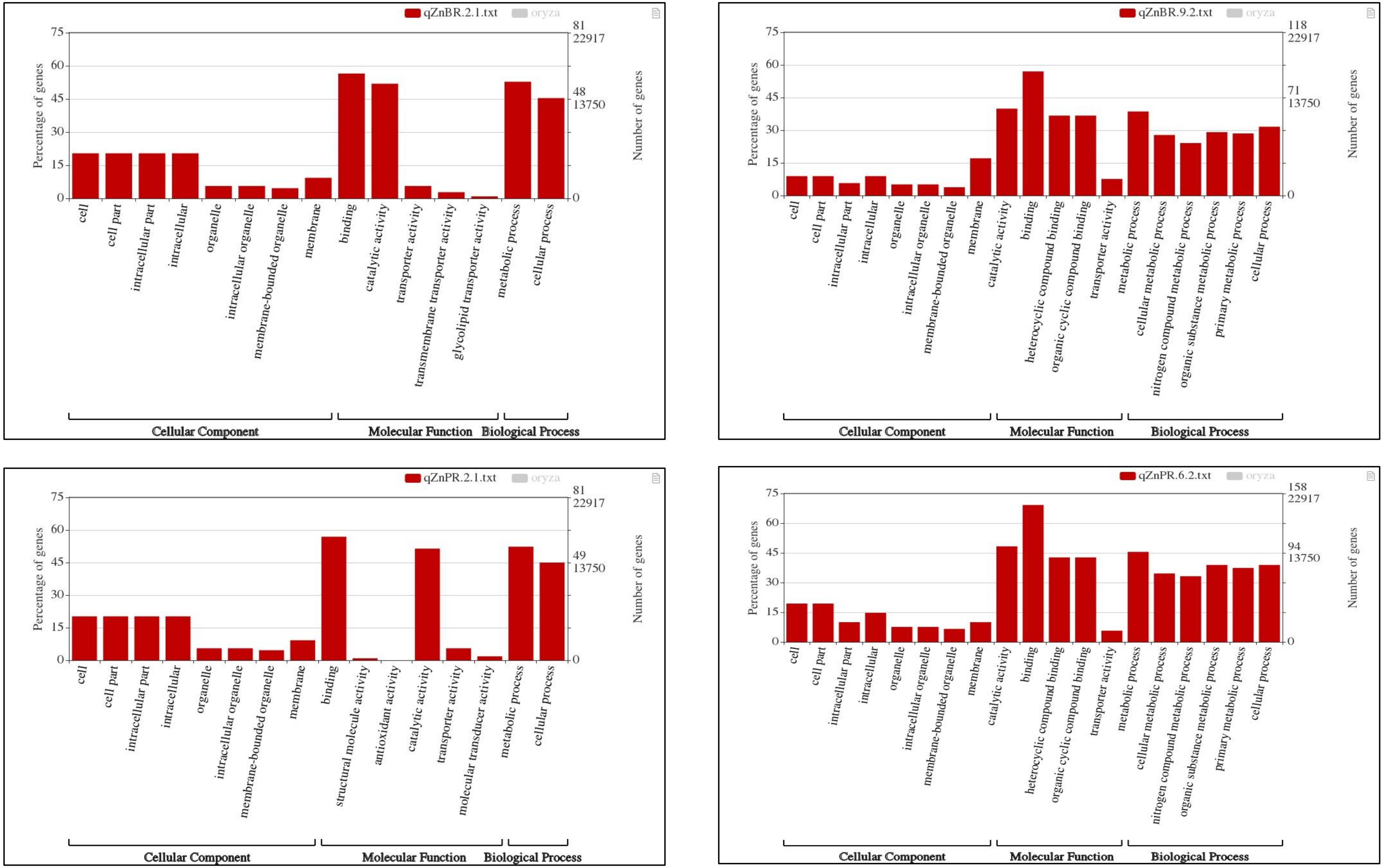

Supplementary Figure S4: Relative expression of (a) Os060705700 and (b) Os06g0706100 in the flag leaf of rice genotypes

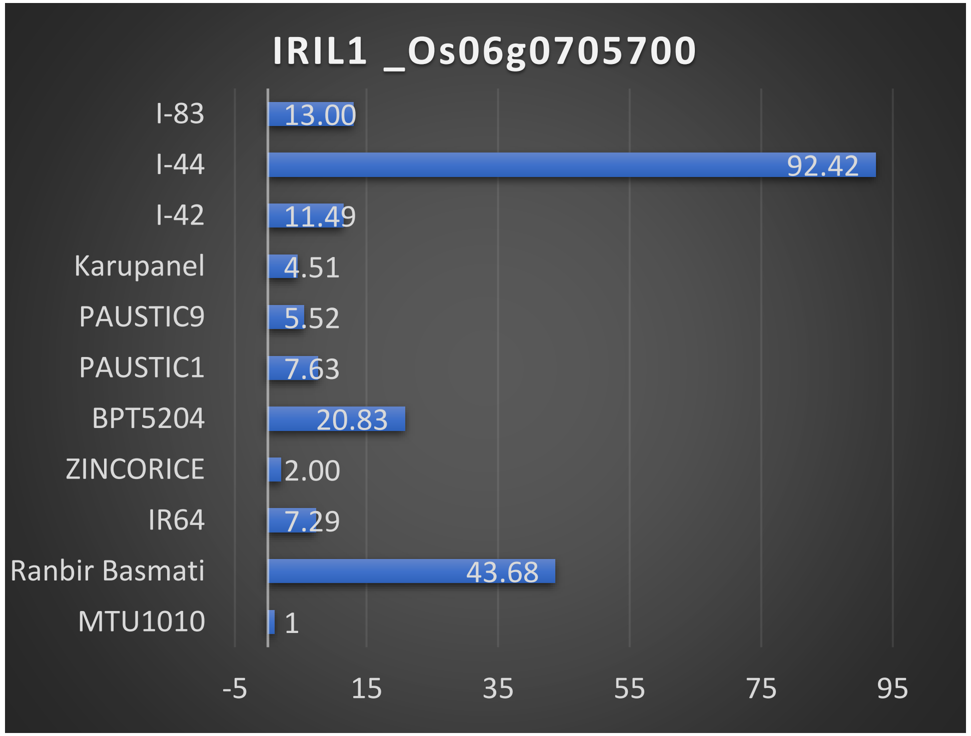

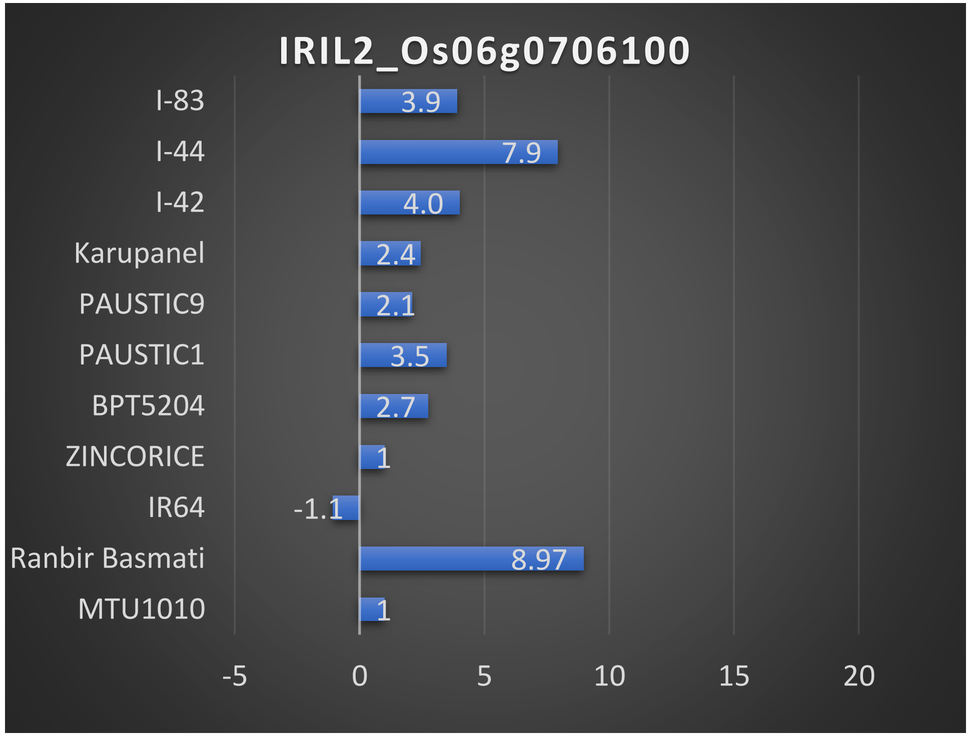

Supplement: Supplementary file 1 [file plants-12-00144-s001.zip › Supplementary Figures.pdf]
